# Complete Genome Sequence of *Edwardsiella tarda* Isolate FL95-01, Recovered from Channel Catfish

**DOI:** 10.1128/genomeA.00682-15

**Published:** 2015-06-25

**Authors:** Stephen R. Reichley, Geoffrey C. Waldbieser, Hasan C. Tekedar, Mark L. Lawrence, Matt J. Griffin

**Affiliations:** a College of Veterinary Medicine, Mississippi State University, Starkville, Mississippi, USA; b USDA-ARS Warmwater Aquaculture Research Unit, Stoneville, Mississippi, USA; c College of Veterinary Medicine, Mississippi State University, Stoneville, Mississippi, USA

## Abstract

*Edwardsiella tarda* is a Gram-negative facultative anaerobe that has been isolated from fish, reptiles, amphibians, and mammals, including humans. This is a report of the complete and annotated genome of isolate FL95-01, recovered from channel catfish (*Ictalurus punctatus*).

## GENOME ANNOUNCEMENT

First recognized in the 1960s (1), *Edwardsiella tarda* is a Gram-negative intracellular facultative anaerobic bacteria infecting a wide range of avian, reptilian, fish, and mammalian hosts across the globe (2–4). Primarily considered a pathogen of marine and freshwater fishes, *E. tarda* has been implicated in considerable economic losses in more than 20 species of commercially important fish worldwide (5). In the catfish-farming region of the southeastern United States, *E. tarda* is associated with emphysematous putrefactive disease of catfish, which begins as small, cutaneous lesions that can progress to deep, malodorous, putrefactive abscesses within the musculature (6, 7). While complete genome sequences for *E. tarda* have been published (8–10), these were later determined to be for *E. piscicida*, a newly adopted taxon in the *Edwardsiella* genus (11–13). The identity of *E. tarda* isolate FL95-01 was confirmed in previous research using repetitive sequence mediated PCR, *gyrB* sequence, and species-specific PCR (13, 14). At present, strain FL95-01 is the only published complete genome sequence for *E. tarda*.

To determine the circularized genome, genomic DNA was sequenced using the Illumina (77× coverage) and Pacific Biosciences (PacBio) (88× coverage) platforms. The longest 40× coverage PacBio reads were error-corrected with the remaining PacBio data using the PBcR module within Celera Assembler version 8.2 beta (15, 16). The longest 25× coverage of the corrected sequence was assembled into a single, circular chromosome. In order to correct variations in homopolymer lengths between PacBio reads, Illumina sequencing reads were mapped to the assembled chromosome with Bowtie2 (17) and the Illumina-preferred consensus sequence was produced using VarScan version 2.3.7 (18).

The circularized and complete genome was submitted to the NCBI Prokaryotic Genomes Annotation Pipeline (PGAP) for annotation and submission to GenBank. Furthermore, the genome was submitted for RAST (19, 20) annotation using the Glimmer option to gather more detailed information.

The *E. tarda* genome consists of one circular chromosome with 3,620,701 bp and 57.3% GC content. PGAP annotation predicted 3,258 genes encoding 3,091 proteins and 101 tRNAs. RAST analysis predicted 505 subsystems with 3,318 coding sequences and 129 RNAs. RAST comparison of FL95-01 with *Edwardsiella piscicida* C07-087 showed 89 unique subsystems in FL95-01 related to carbohydrate metabolism, cell wall and capsule, and copper tolerance. Additionally, RNAmmer (21) predicted 9 rRNA operons. FL95-01 does not carry any plasmids. Average nucleotide identities (ANI), as calculated by Goris et al. (22), were estimated using an online calculator (http://enve-omics.ce.gatech.edu/ani). The complete genome of *E. tarda* isolate FL95-01 shares an ANI of 83.4% with *E. piscicida* isolate C07-087 (GenBank accession no. CP004141) (10), 83.9% with *E. piscicida* isolate 080813 (GenBank accession no. CP006664) (23), and 83.1% with *E. ictaluri* isolate 93-146 (GenBank accession no. CP001600) (24). 

### Nucleotide sequence accession number.

The complete genome sequence for *Edwardsiella tarda* isolate FL95-01 has been deposited in GenBank under the accession number CP011359.
